# Body Fluid Interferon-γ Release Assay for Diagnosis of Extrapulmonary Tuberculosis in Adults: A Systematic Review and Meta-Analysis

**DOI:** 10.1038/srep15284

**Published:** 2015-10-27

**Authors:** Xiao-Xia Zhou, Ya-Lan Liu, Kan Zhai, Huan-Zhong Shi, Zhao-Hui Tong

**Affiliations:** 1 Department of Respiratory and Critical Care Medicine, Beijing Chao-Yang Hospital, Capital Medical University, Beijing, China; 2 Geriatric Department, Fu Xing hospital, Capital Medical University, Beijing, China; 3 Medical Research Center, Beijing Chao-Yang Hospital, Capital Medical University, Beijing, China

## Abstract

The diagnosis of extrapulmonary tuberculosis (EPTB) is difficult. In recent years, T-cell interferon-γ release assays (IGRAs) are widely used in diagnosing tuberculosis. The aim of this meta-analysis is to evaluate the diagnostic accuracy of body fluid IGRAs in diagnosing EPTB. The PubMed, EMBASE, Web of Science, and Cochrane bibliographies were searched for English language articles. 22 studies met the inclusion criteria. The pooled sensitivity and specificity of body fluid IGRAs for diagnosing EPTB were 0.87 [95% confidence interval (CI): 0.83–0.92] and 0.85 (95% CI: 0.79–0.90), respectively. For the fluid T-SPOT.TB, the pooled sensitivity and specificity were 0.92 (95% CI: 0.88–0.95) and 0.85 (95% CI: 0.78–0.91), respectively. The diagnostic odds ratio (DOR) of the fluid T-SPOT.TB was 46.99 (95% CI: 13.69–161.28) for tuberculosis pleurisy, 26.46 (95% CI: 11.38–61.56) for tuberculosis peritonitis, and 97.86 (95% CI: 25.31–378.45) for tuberculosis meningitis. The application of T-SPOT. TB in the diagnosis of EPTB performed better in the body fluid than in the blood. The diagnostic values of the fluid T-SPOT.TB varied for different fluid categories. However, the utility of T-SPOT.TB was limited due to its suboptimal accuracy and higher cost compared with conventional tests.

Tuberculosis (TB) is a serious global public health problem and a leading cause of morbidity and mortality throughout the world. Extrapulmonary tuberculosis (EPTB) includes meningitis, genitourinary infection, pericarditis, lymphadenitis, pleurisy, peritonitis, musculoskeletal infection, and cutaneous tuberculosis. In 2012, 6.1 million cases of TB were notified, and the prevalence of EPTB was approximately 13.1% (ranged 0.7%–38.0%)^1^. However, the manifestation of EPTB was highly heterogeneous. Delayed diagnosis contributes significantly to morbidity and mortality.

Rapid diagnosis and treatment is crucial for the effective control of TB in clinical practice in EPTB patients. Mycobacterial culture of the body fluid or biopsy specimens is considered the gold standard for the diagnosis of EPTB. However, the obtained fluid sample may be paucibacillary, the mycobacterial culture requires a long period of time, and the diagnostic yield of effusion is only 63%^2^. The cell infiltrate profile, microbiological examination, adenosine deaminase (ADA) level, and other biochemical tests of pleural effusion lack sensitivity and specificity^3^. Although diagnosis can also be established by invasive procedures, such approaches place patients at an increased risk of complications and result in higher costs^4^. Therefore, a faster, more sensitive, and specific test for the diagnosis of EPTB in routine clinical practice is required.

Recently developed interferon-γ release assays (IGRAs) are sensitive, specific, and rapid immunodiagnostic tests for TB infection. They detect interferon-γ (IFN-γ) produced by lymphocytes in response to *Mycobacterium tuberculosis* (MTB)-specific antigens, early secretory antigenic target 6 (ESAT-6), and culture filtrate protein-10 (CFP-10). Two commercial tests are available: the T-SPOT.TB (Oxford Immunotec, Abingdon, UK), which measures the number of IFN-γ-producing T cells by enzyme-linked immunospot (ELISPOT) assay, and the QuantiFERON-TB Gold In-Tube (QFT-GIT) test and its predecessor the QuantiFERON-TB Gold (QFT-G) test (Cellestis Ltd., Carnegie, Australia), which detect IFN-γ in culture supernatant by enzyme-linked immunosorbent assay (ELISA). One of the theoretical advantages of blood IGRAs over TST is their higher specificity, because IGRAs do not cross-react with the Bacillus of Calmette and Guérin (BCG) vaccine antigens^5^. They cannot distinguish latent TB infection (LTBI) from active TB^6^, and their applications in high-TB-burden countries are limited. It was hypothesized that *M. tuberculosis* antigen-specific T cells may accumulate at infection sites. Therefore, in EPTB, IGRAs of body fluid samples from infection sites may increase diagnostic specificities.

Recent studies mainly focused on blood IGRAs and reported suboptimal results in diagnosing EPTB^7^. Some also investigated body fluid IGRAs for diagnosing EPTB^8^,^9^,^10^. This meta-analysis was performed to establish the overall accuracy of body fluid and blood IGRAs for diagnosing EPTB, and to assess the diagnostic value of the body fluid T-SPOT.TB from different fluid sites.

## Results

### Characteristics of the studies

**Figure 1:**
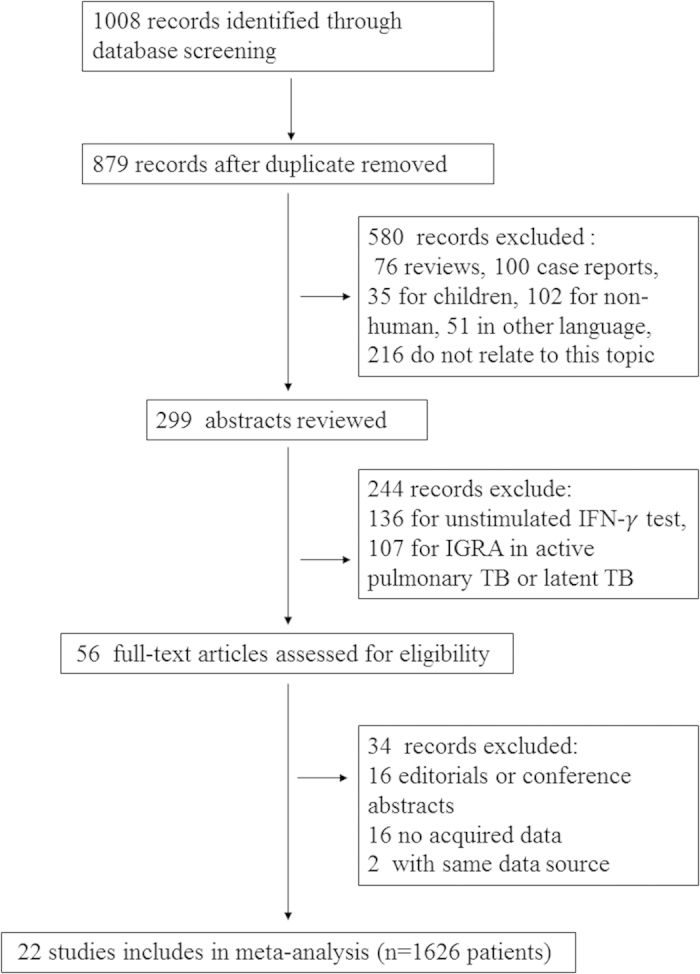
Flow diagram for study selection. Abbreviations: IGRA, interferon-γ release assay; *n*, number of participants.

A total of 1008 citations were found for patients with tuberculosis diagnosed by IGRAs (Fig. 1). After independent reviews, 22 studies^11^,^12^,^13^,^14^,^15^,^16^,^17^,^18^,^19^,^20^,^21^,^22^,^23^,^24^,^25^,^26^,^27^,^28^,^29^,^30^,^31^,^32^ on EPTB with commercial IGRAs using the body fluid met the inclusion criteria (*n *= 1626; Fig. 1 and supplementary Table S1). Only one study^17^ did not provide blood IGRA results (supplementary Table S2). The ELISPOT-based T-SPOT.TB tests were performed in 15 studies^11^,^12^,^13^,^14^,^15^,^20^,^21^,^22^,^23^,^24^,^25^,^28^,^29^,^30^,^32^, and the ELISA-based QFT-G or QFT-GIT assays were used in 9 studies^15^,^16^,^17^,^18^,^19^,^25^,^26^,^27^,^31^. Head-to-head comparisons of the diagnostic accuracies of T-SPOT.TB against QFT-GIT were found in two studies^15^,^25^. In one study^27^, the probable cases were excluded in the final analysis because a follow-up examination of the anti-TB treatment response was not performed. Studies with sample sizes of less than 10 patients were excluded from the final analysis^27^,^32^. The characteristics of the 22 eligible studies, including country of origin, indeterminate result, human immunodeficiency virus (HIV) infection condition, IGRAs method, cutoff value, and study quality are presented in supplementary Tables S1 and S2.

Confirmed cases were available in nine studies^12^,^13^,^20^,^21^,^24^,^25^,^29^,^30^,^32^ for T-SPOT.TB and six studies^16^,^17^,^18^,^25^,^27^,^31^ for ELISA. Some data from three studies were excluded for limited sample size^27^,^28^,^32^.

The numbers of patients were extracted from nine studies on tuberculous pleurisy^11^,^12^,^13^,^14^,^15^,^23^,^25^,^30^,^32^, three studies on peritonitis^11^,^20^,^24^, and five studies on meningitis^21^,^22^,^23^,^28^,^29^. The diagnostic value of the fluid T-SPOT.TB in pericarditis^11^ was not analyzed for the limited sample size. The diagnostic accuracies of the fluid T-SPOT.TB were compared with the nonspecific biochemical test based on previous meta-analyses according to fluid categories. Those tests included pleural fluid ADA^33^, pleural fluid unstimulated IFN-γ^34^, peritoneal fluid ADA^35^, and cerebrospinal fluid ADA^36^.

Nine studies were performed in non-HIV patients^11^,^12^,^13^,^15^,^18^,^24^,^28^,^31^,^32^, four studies^16^,^17^,^19^,^30^ did not report the HIV status, and the remaining nine studies^14^,^20^,^21^,^22^,^23^,^25^,^26^,^27^,^29^ contained HIV patients ranging from 1.1% to 87.1% of the study cohort.

Indeterminate results were recorded in 11 studies^11^,^13^,^14^,^17^,^19^,^20^,^21^,^22^,^25^,^28^,^29^, including a total of 162 cases, and indeterminate rates were pooled.

### Quality of the studies

Only one study^32^ had a Standards for Reporting of Diagnostic Accuracy (STARD) score less than 13. Eight^13^,^15^,^16^,^20^,^23^,^24^,^26^,^29^ out of 22 studies had Quality Assessment of Studies of Diagnostic Accuracy included in Systematic Reviews (QUADAS) scores greater than 10, and were considered as higher quality. The STARD and QUADAS scores are outlined in supplementary Tables S1 and S2.

### Pooled sensitivity and specificity in the body fluid and peripheral blood by ELISPOT and ELISA

**Figure 2:**
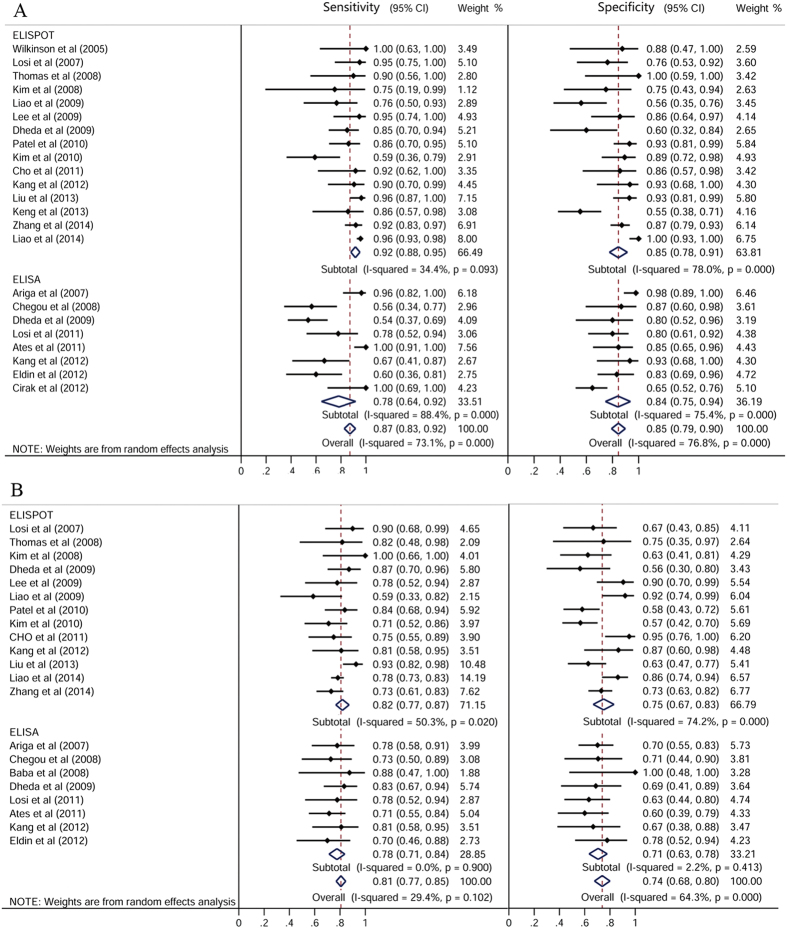
Forest plot showing estimates of sensitivities and specificities for T-cell interferon-γ release assays in the (**A**) body fluid and (**B**) peripheral blood for the diagnosis of EPTB. The solid dots represent the point estimates of sensitivity and specificity from each study. Error bars indicate 95% CIs. Pooled results are shown as diamonds.

The pooled sensitivities for IGRAs were 0.87 [95% confidence interval (CI): 0.83–0.92] in the body fluid and 0.81 (95% CI: 0.77–0.85) in the peripheral blood (Fig. 2). The pooled specificities of IGRAs were 0.85 (95% CI: 0.79–0.90) in the body fluid and 0.74 (95% CI: 0.68–0.80) in the blood. The positive likelihood ratio (PLR), negative likelihood ratio (NLR), and diagnostic odds ratio (DOR) of body fluid IGRAs were 5.02 (95% CI: 3.36–7.52), 0.17 (95% CI: 0.11–0.28), and 35.19 (95% CI: 17.83–69.47), respectively. While the PLR, NLR, and DOR of blood IGRAs in the diagnosis of EPTB were 2.58 (95% CI: 2.16–3.08), 0.30 (95% CI: 0.26–0.35), and 9.89 (95% CI: 7.14–13.70), respectively.

**Figure 3:**
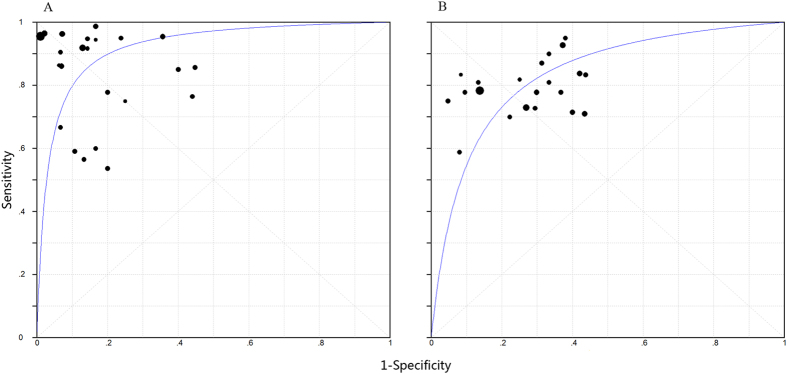
Summary receiver operating characteristic (SROC) curves for T-cell interferon-γ release assays in the (**A**) body fluid and (**B**) peripheral blood. Each study included in the meta-analysis is shown as a solid circle. The size of each study is indicated by the size of the solid circle. The regression SROC curves summarize the overall diagnostic accuracy.

Figure 3 shows the summary receiver operating characteristic (SROC) curves for IGRAs from individual studies. The SROC curve for blood IGRAs was not positioned near the desirable upper left corner; the best joint sensitivity and specificity was 0.767 and the area under the curve (AUC) was 0.835 [standard error of the mean (SEM) = 0.018] (Fig. 3B). The optimum joint sensitivity and specificity of body fluid IGRAs was 0.856 with an AUC of 0.922 (SEM = 0.019) (Fig. 3A).

The pooled sensitivities for T-SPOT.TB and QFT-G or QFT-GIT in the body fluid were 0.92 (95% CI: 0.88–0.95) and 0.78 (95% CI: 0.64–0.92), respectively. The pooled specificities for T-SPOT.TB and QFT-G or QFT-GIT in the body fluid were 0.85 (95% CI: 0.78–0.91) and 0.84 (95% CI: 0.75–0.94), respectively (Fig. 2).

### Subanalysis conducted including only confirmed cases

The meta-analytic estimate of sensitivity, specificity, and AUC of T-SPOT.TB were 0.95, 0.88, and 0.97, respectively, for the body fluid, and 0.82, 0.70, and 0.88, respectively, for the blood. And sensitivity, specificity, and AUC of QFT-GIT were 0.78, 0.75, and 0.91, respectively, for the body fluid, and 0.79, 0.71, and 0.83, respectively, for the blood. Details are shown in Table 1.

### Subanalysis for the body fluid T-SPOT.TB sensitivity and specificity in serous fluids from different sites

In this subanalysis, the DOR of the body fluid T-SPOT.TB was 96.86 in patients with tuberculous peritonitis and 26.46 in patients with tuberculous meningitis (Table 2). The sensitivity of T-SPOT.TB was 0.94 (95% CI: 0.91–0.96) for pleurisy, 0.94 (95% CI: 0.83–0.99) for peritonitis, and 0.75 (95% CI: 0.64–0.84) for meningitis. And the specificity was 0.80 (95% CI: 0.75–0.85) for pleurisy, (0.87, 95% CI: 0.76–0.94) for peritonitis, and 0.91 (95% CI: 0.83–0.96) for meningitis.

### Diagnostic accuracy comparison of the body fluid T-SPOT.TB with ADA and unstimulated IFN-γ in different fluid categories

In tuberculosis pleurisy, the sensitivity, specificity, PLR, NLR, and DOR were 0.94, 0.80, 4.83, 0.11, and 46.99 for the pleural fluid T-SPOT.TB; 0.92, 0.90, 9.03, 0.10, and 110.08 for the pleural fluid ADA^33^; and 0.89, 0.97, 23.45, 0.11, and 272.7 for the pleural fluid unstimulated IFN-γ^34^, respectively (Table 2).

The sensitivity, specificity, PLR, and NLR were 0.94, 0.87, 6.93, and 0.07 for the peritoneal fluid T-SPOT.TB and 1.00, 0.97, 26.8, and 0.038 for the peritoneal fluid ADA^35^, respectively.

In tuberculous meningitis, the sensitivity, specificity, PLR, NLR, and DOR were 0.75, 0.91, 8.02, 0.29, and 26.46, respectively, for the cerebrospinal fluid (CSF) T-SPOT.TB and 0.79, 0.91, 6.85, 0.29, and 26.92, respectively, for the CSF ADA^32^ (Table 2).

The AUC of the fluid T-SPOT.TB was 0.97, 0.93, and 0.93 for pleurisy, peritonitis, and meningitis, respectively.

### Pooled IGRA sensitivity and specificity in high- and low-TB-burden countries

**Figure 4:**
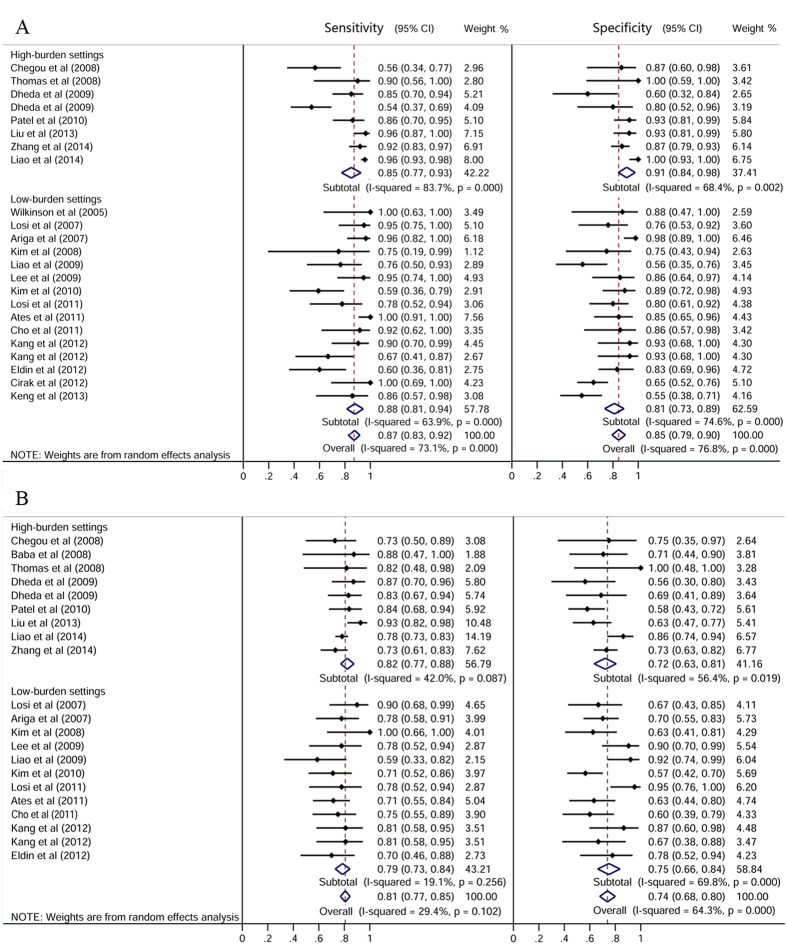
Sensitivity and specificity for T-cell interferon-γ release assays in the (**A**) body fluid and (**B**) peripheral blood for the diagnosis of EPTB, stratified by tuberculous burden settings. The solid dots represent the point estimates of sensitivity and specificity from each study. Error bars indicate 95% CIs. Pooled results are shown as diamonds.

In high-burden settings, fluid IGRAs had a sensitivity of 0.85 (95% CI: 0.77–0.93) and a specificity of 0.91 (95% CI: 0.84–0.98). In low-burden settings, the sensitivity of fluid IGRAs was 0.88 (95% CI: 0.81–0.94) and specificity was 0.81 (95% CI: 0.73–0.89) (Fig. 4). For the fluid T-SPOT.TB, in high-burden settings, the sensitivity and specificity were 0.94 (95% CI: 0.91–0.97) and 0.92 (95% CI: 0.85–0.99), respectively. In low-burden settings, the sensitivity and specificity of the fluid T-SPOT.TB were 0.88 (95% CI: 0.81–0.96) and 0.79 (95% CI: 0.69–0.88), respectively.

### Pooled fluid T-SPOT.TB sensitivity and specificity in non-HIV group

In non-HIV studies that used the fluid T-SPOT.TB, the sensitivity for developing EPTB was 0.95 (95% CI: 0.93–0.97), specificity was 0.91 (95% CI: 0.87–0.95), and the DOR was 163.53 (95% CI: 82.536–324.01).

### Indeterminate results

The indeterminate results for the fluid T-SPOT.TB was 81 (*n *= 1130, 6.2%) and that for the blood T-SPOT.TB was 19 (*n *= 1091, 1.4%). The pooled indeterminate rates were 0.06 (95% CI: 0.05–0.08) for the fluid T-SPOT.TB and 0.04 (95% CI: 0.02–0.05) for the blood T-SPOT.TB. For QFT-GIT, the indeterminate results in the fluid test was 47 (*n *= 463, 10.1%) and that in the blood test was 15 (*n *= 377, 4.0%). The pooled indeterminate rates were 0.15 (95% CI: 0.10–0.19) for the fluid QFT-GIT and 0.09 (95% CI: 0.04–0.14) for the blood QFT-GIT.

### Meta-regression analysis and publication bias

The QUADAS score was used to evaluate the effect of study quality on the relative DOR of IGRAs for the diagnosis of EPTB by meta-regression analysis. The local TB burden condition, fluid category, and assay method were also assessed (Table 3).

The funnel plots for publication bias showed some asymmetry (supplementary Fig. S1). Publication bias was significant for blood IGRAs (*P* = 0.003) but not for fluid IGRAs (*P *= 0.203) by the Egger test. No publication bias was found in both the fluid (*P *= 0.411) and the blood (*P *= 0.606) QFT-GIT studies.

## Discussion

Data on the diagnostic value of IGRAs in the body fluid are accumulating. In this meta-analysis, the T-SPOT.TB was found to be used for the diagnosis of EPTB in patients with pleurisy, pericarditis, peritonitis, and meningitis, while the QFT-GIT test was mainly applied in tuberculous pleurisy. Few meta-analyses have examined the performance of body fluid IGRAs in EPTB^8^,^9^,^10^. The aim of this study was to evaluate the diagnostic accuracy of fluid IGRAs in EPTB, and to assess the specific value of the fluid T-SPOT.TB in different fluid category.

This study reported pooled sensitivities and specificities of 0.92 and 0.85 for the fluid T-SPOT.TB, and 0.78 and 0.84 for the fluid QFT-GIT, respectively. In confirmed cases, both sensitivity and specificity of the fluid T-SPOT.TB (0.95 and 0.88) were higher than those of the fluid QFT-GIT (0.78 and 0.75), in agreement with another meta-analysis that evaluated extrasanguinous specimens^10^. The pooled sensitivity, specificity, and AUC of the fluid T-SPOT.TB in confirmed cases also showed some advantage over those of the blood T-SPOT.TB, which complied with the conclusion of a previous meta-analysis on tuberculous pleurisy^8^. These results support that *M. tuberculosis* antigen-specific T cells would accumulate at infection sites in active tuberculosis^29^,^30^. In summary, the fluid T-SPOT.TB appeared to be the best immunodiagnostic test in diagnosing EPTB.

The diagnostic accuracy of the fluid T-SPOT.TB varied with the fluid category. The DOR of T-SPOT.TB with pleural fluid tended to be higher compared with CSF and lower compared with peritoneal fluid, but all the differences were not significant. The T-SPOT.TB sensitivities and specificities in patients with pleurisy and peritonitis were similar. However, the sensitivity was significantly lower in the CSF T-SPOT.TB than in the pleural fluid T-SPOT.TB (0.75 vs 0.94). One possible reason for the low sensitivity of the T-SPOT.TB assay in tuberculosis meningitis may be the low antigenic loading and severe disease manifestation early in the progression of tuberculosis meningitis^22^.

However, the overall accuracy of the fluid T-SPOT.TB still showed no advantage over the body fluid ADA level analysis and pleural IFN-γ analysis. Nearly 20% of patients are misdiagnosed with tuberculosis pleurisy by T-SPOT.TB. For tuberculosis peritonitis, almost 13% non-TB patients would be incorrectly treated for tuberculosis. When diagnosed with fluid ADA, there would be about 10% and 3% of patients misdiagnosed with tuberculous pleurisy and tuberculous peritonitis, respectively. Additionally, in TB meningitis, 25% TB patients would be missed. Although the utility of the CSF ADA was also limited due to its low sensitivities, however, because the CSF ADA was convenient and inexpensive and had a specificity of 0.91 (95% CI: 0.89–0.93), it might assist in excluding tuberculous meningitis during differential diagnosis. Altogether, considering the lower cost and easier accessibility of the fluid ADA and the pleural fluid IFN-γ, IGRAs showed no obvious advantage in clinical application. However, because the sample size was much smaller in the peritonitis group, further studies are needed.

Hypothetically, for blood IGRAs, the specificity should be lower in high-burden settings due to the high latent TB rate than in low-burden settings. However, in this study, no significant difference was found in both specificity and sensitivity of fluid and blood IGRAs between different TB-burden settings. Even more, the specificity of the fluid T-SPOT.TB appeared to be higher in high-burden settings than in low-burden settings. This finding suggests that the LTBI condition may have less influence on IGRAs, especially in fluid samples. The performance of the fluid T-SPOT.TB generates 6% false-negative and 8% false-positive results for EPTB diagnosis in high-burden settings and is not superior to the fluid ADA analysis.

The HIV-associated immunocompromised state may weaken the ability of IGRAs to detect tuberculosis infection^37^. In the present research, three studies found equal performance of fluid IGRAs in both HIV and non-HIV patients, but higher indeterminate results were found in the HIV group^25^,^26^,^27^. A subanalysis of the fluid T-SPOT.TB assay was performed in HIV-negative patients, an insignificant elevation was found in sensitivity and specificity compared with the pooled fluid T-SPOT.TB. While a recent systemic review found that patients with a CD4 cell count <200 cells/mm^3^ present with suboptimal accuracy of blood IGRAs for diagnosing active tuberculosis disease^37^, another study^38^ found similar test accuracy for the blood T-SPOT.TB regardless of whether the patient’s CD4 cell count was higher/equal or lower than 200 cells/mm^3^. Whether HIV status or CD4 cell count may have influence on the accuracy of fluid IGRAs still lacks evidence.

Identifying the cause of uninterpretable results is important. Most of the indeterminate results were due to a high nil control. The proportion of indeterminate results was higher in the QFT-GIT test than in the T-SPOT.TB assay. In some studies, the indeterminate results of QFT-GIT could be reduced through sample dilution^17^,^19^, indicating that high backgrounds may result from large amount of cells, proteins, and fibrins in supernatants. Fluid samples appeared to correspond with higher percentages of invalid results, and in two studies^15^,^21^, the indeterminate results from the fluid T-SPOT.TB diminished after a new cutoff value was used. Some indeterminate results were due to a weak response in the positive control well. Researchers consider this to be a reflection of old age or an immunocompromised condition, such as HIV infection^14^,^15^,^37^. However, disagreement also exists^39^. The cause of a low positive control in T-SPOT.TB is still controversial. Moreover, whether the technical parameters for blood IGRAs, such as time of incubation, are suitable for fluid IGRAs is still unknown. Hence, no widely accepted standard hindered the interpretation of the test results of fluid IGRAs.

This study had some limitations. First, publication bias may have been present due to the exclusion of conference abstracts, letters, and, particularly, non-English articles, as TB is more prevalent in non-English speaking countries. Second, the validity of the results is weakened by the inconsistencies across the studies. Heterogeneity was still present after performing subgroup analyses. Furthermore, because this meta-analysis was restricted to studies in adults, the utility of IGRAs in children was ignored, although they are the major sufferers for EPTB. The exclusion of indeterminate results in analyzing the diagnostic accuracy of IGRAs would cause inaccurate estimates in practical applications, and the confirmed cases were not true confirmation, because not all cases were diagnosed with the positive culture or nucleic acid amplification test (NAAT) of *M. tuberculosis*.

The overall performances of fluid and blood IGRAs for the diagnosis of tuberculosis were not as high as expected and varied by fluid category. The indeterminate result rates were high in fluid IGRAs. The fluid T-SPOT.TB was the best immunodiagnostic test for EPTB, and showed a better performance in high-burden settings, but was not remarkably superior to the fluid ADA test. With regard to its high cost, sophisticated technique, and difficulty in interpretation, the application of fluid IGRAs was limited in diagnosing EPTB.

## Methods

### Publication search

A systematic search of studies evaluating the diagnostic value of body fluid IGRAs in patients with EPTB was carried out. All English studies performed were searched on human subjects published until April 26, 2014, in electronic databases, including PubMed, EMBASE, Web of Science, and Cochrane-controlled central register of controlled trials. A lower date limit was not applied. The following search terms were used: “tuberculous,” “tuberculosis,” “fluid,” “effusion,” “interferon-γ,” “t-spot,” and “quantiferon.” The research strategy also contained text terms, such as “pleural,” “pericardial,” “peritoneal,” and “cerebral.” Citations in these articles were also searched.

All the abstracts were read to select the appropriate studies on body fluid samples in extrapulmonary tuberculosis (EPTB) patients with full texts available online. Studies using noncommercial IGRAs or presenting nonoriginal data were excluded, as were conference abstracts, editorials, reviews, guidelines, animal studies, and studies conducted in children.

### Data extraction and quality assessment

Two investigators (L. YL and Z. XX) independently extracted data from the selected studies using a data extraction form designed before beginning the study, and the methodological qualities of the studies were assessed independently. Differences were resolved by consensus or a third investigator (S. HZ).

This meta-analysis was conducted in accordance with the guidelines of the preferred reporting items for systematic reviews and meta-analyses (PRISMA) statement^40^. All selected studies were evaluated by standard methods recommended for diagnostic meta-analysis^41^. The qualities of the studies were assessed with both the Standards for Reporting of Diagnostic Accuracy (STARD) score^42^ (maximum score 25) and the Quality Assessment of Studies of Diagnostic Accuracy included in Systematic Reviews (QUADAS) score^43^ (maximum score 14) checklist. Ethical approval was not needed for this study.

### Data synthesis

EPTB was defined when an *M. tuberculosis* culture was positive, a nucleic acid amplification test (NAAT) was positive, and/or histopathological examination revealed the presence of caseous necrosis with or without acid-fast bacilli, and/or granuloma formation. Patients were classified as probable EPTB, if MTB could not be identified from the aforementioned tests, and positive treatment responses to a full course of anti-TB therapy should be observed. Because no standard cutoff value for body fluid IGRAs are available, the results were analyzed with the best diagnostic accuracy, if provided, and the cutoff value recorded. Indeterminate results were excluded from further analysis. The results were grouped according to the tuberculosis burden reported by the World Health Organization (WHO)^1^. A comparison of serous fluid and whole blood IGRA assays were made. And subanalyses were performed in confirmed cases, different fluid categories, low- and high-TB-burden settings, non-HIV population, and indeterminate results.

### Statistical analysis

Two statistical software programs were used for the analyses: Stata (version 12; Stata Corporation, TX, USA) and Meta-Disc for Windows (XI Cochrane Colloquium, Barcelona, Spain). The following outcomes for each study were assessed and pooled when feasible: sensitivity, specificity, positive likelihood ratio (PLR), negative likelihood ratio (NLR), diagnostic likelihood ratio (DOR), and rates of indeterminate results. Heterogeneity across studies was detected by Chi-square-based Q test and Fisher’s exact tests, and publication bias was assessed by funnel plots and the Egger test^44^. When *I*^2^ < 50% or *P *> 0.05 in *Q* test, a fixed-effect model (Mantel–Haenszel method) was used to evaluate the pooled sensitivity, specificity, PLR, NLR, and DOR. Otherwise, a random-effect model (DerSimonian–Laird method) was used. While comparing method A with method B, if the mean for method A was higher than the mean for method B without overlap of 95% CI, then A was better than B. A two-sided *P* value < 0.05 was considered statistically significant.

## Additional Information

**How to cite this article**: Zhou, X.-X. *et al.* Body Fluid Interferon-γ Release Assay for Diagnosis of Extrapulmonary Tuberculosis in Adults: A Systematic Review and Meta-Analysis. *Sci. Rep.* **5**, 15284; doi: 10.1038/srep15284 (2015).

## Supplementary Material

Supplementary Information

## Figures and Tables

**Table 1:** Pooled results for diagnostic accuracy of interferon-γ release assays in patients with confirmed extrapulmonary tuberculosis.

	Body Fluid			Peripheral Blood		
	Total	ELISPOT	ELISA	Total	ELISPOT	ELISA
Number of studies	12	8	5	11	7	4
Sensitivity (95% CI)	0.91 (0.89–0.92)	0.95 (0.92–0.96)	0.78 (0.69–0.85)	0.81 (0.78–0.85)	0.82 (0.78–0.85)	0.79 (0.67–0.88)
Heterogeneity* (P)	67.14 (<0.001)	11.58 (0.115)	31.06 (<0.001)	16.74 (0.080)	14.44 (0.025)	1.89 (0.595)
Specificity (95% CI)	0.82 (0.78–0.86)	0.88 (0.83–0.92)	0.75 (0.68–0.81)	0.70 (0.65–0.75)	0.70 (0.64–0.76)	0.71 (0.61–0.80)
Heterogeneity (P)	70.56 (<0.001)	27.94 (<0.001)	30.53 (<0.001)	22.40 (0.013)	17.69 (0.007)	4.68 (0.197)
PLR (95% CI)	5.11 (2.80–9.32)	6.23 (2.85–13.64)	3.45 (1.70–6.98)	2.71 (2.18–3.37)	2.82 (2.06–3.88)	2.62 (1.89–3.63)
Heterogeneity (P)	86.05 (<0.001)	33.87 (<0.001)	20.91 (<0.001)	14.64 (0.146)	13.33 (0.038)	1.5 (0.683)
NLR (95% CI)	0.11 (0.05–0.25)	0.085 (0.05–0.16)	0.19 (0.05–0.67)	0.25 (0.21–0.31)	0.24 (0.19–0.30)	0.31 (0.20–0.49)
Heterogeneity (P)	85.65 (<0.001)	12.93 (0.074)	20.91 (<0.001)	6.27 (0.792)	4.6 (0.596)	1.24 (0.744)
DOR (95% CI)	57.83 (20.13–166.16)	91.73 (26.75–314.56)	27.89 (4.78–162.86)	13.98 (9.22–21.20)	16.40 (9.97–26.99)	9.65 (4.52–20.60)
Heterogeneity (P)	37.28 (<0.001)	17.18 (0.016)	13.50 (0.009)	7.01 (0.724)	4.51 (0.608)	1.18 (0.758)
AUC (SEM)	0.95 (0.022)	0.97 (0.013)	0.91 (0.066)	0.86 (0.020)	0.88 (0.022)	0.83 (0.043)

^*^Chi-square value.

Abbreviations: AUC, area under the curve; DOR, diagnostic odds ratio; ELISA, enzyme-linked immunosorbent assay; ELISPOT, enzyme-linked immunosorbent spot; NLR, negative likelihood ratio; PLR, positive likelihood ratio.

**Table 2:** Diagnostic value of the body fluid T-SPOT.TB, ADA and unstimulated IFN-γ based on fluid category.

	Pleurisy		Peritonitis		Meningitis		
	T-SPOT.TB	ADA	IFN-γ	T-SPOT.TB	ADA	T-SPOT.TB	ADA
Number of studies (N)	9 (744)	63 (8093)	22 (2101)	3 (116)	4 (264)	5 (173)	10 (1472)
Sensitivity (95% CI)	0.94 (0.91–0.96)	0.92 (0.90–0.93)	0.89 (0.87–0.91)	0.94 (0.83–0.99)	1.00 (0.93–1.00)	0.75 (0.64–0.84)	0.79 (0.75–0.83)
Heterogeneity* (P)	11.29 (0.186)	327.03 (<0.001)	67.54 (<0.001)	0.13 (0.938)	0.0 (1.000)	9.54 (0.049)	44.80 (<0.001)
Specificity (95% CI)	0.80 (0.75–0.85)	0.90 (0.89–0.91)	0.97 (0.96–0.98)	0.87 (0.76–0.94)	0.97 (0.94–0.99)	0.91 (0.83–0.96)	0.91 (0.89–0.93)
Heterogeneity (P)	62.19 (<0.001)	303.79 (<0.001)	32.66 (0.050)	0.05 (0.977)	1.14 (0.768)	5.41 (0.248)	63.60 (<0.001)
PLR (95% CI)	4.83 (2.00–11.66)	9.03 (7.19–11.35)	23.45 (17.31–31.78)	6.93 (3.75–12.80)	26.8 (13.3–54.0)	8.02 (4.19–15.37)	6.85 (4.11–11.41)
Heterogeneity (P)	90.45 (<0.001)	380.14 (<0.001)	21.21 (0.446)	0.05 (0.973)	0.16 (0.984)	4.13 (0.389)	51.66 (<0.001)
NLR (95% CI)	0.11 (0.06–0.20)	0.10 (0.07–0.14)	0.11 (0.07–0.16)	0.07 (0.02–0.21)	0.038 (0.01–0.15)	0.29 (0.20–0.42)	0.29 (0.19–0.44)
Heterogeneity (P)	19.20 (0.014)	381.73 (<0.001)	62.36 (<0.001)	0.14 (0.931)	0.24 (0.9)	11.25 (0.024)	34.82 (<0.001)
DOR (95% CI)	46.99 (13.69–161.28)	110.08 (69.96–173.20)	272.7 (147.5–504.2)	97.86 (25.31–378.45)	–	26.46 (11.38–61.56)	26.92 (12.72–56.97)
Heterogeneity (P)	28.43 (<0.001)	291.60 (<0.001)	30.04 (0.091)	0.13 (0.938)	–	4.91 (0.297)	29.75 (<0.001)
AUC (SEM)	0.97 (0.012)	0.96 (–)	0.99 (–)	0.93 (0.161)	0.99 (0.00)	0.93 (0.045)	0.92 (–)

^*^Chi-square value.

Abbreviations: AUC, area under the curve; DOR, diagnostic odds ratio; N, number of participants; NLR, negative likelihood ratio; PLR, positive likelihood ratio.

**Table 3:** Weighted meta-regression of the effects of study settings, methods, and methodological quality on diagnostic accuracy of interferon-γ release assays.

Covariate	Number of studies	Co-efficient	RDOR	P-value
**Body Fluid**				
QUADAS score				
≥11	8	–0.46	0.63 (0.14–2.92)	0.537
<11	13			
Setting				
High TB burden	8	–0.41	0.67 (0.14–3.13)	0.591
Low TB burden	15			
Method				
ELISPOT	15	–0.44	0.64(0.13–3.09)	0.437
ELISA	8			
Disease				
Pleurisy	14	–0.43	0.65 (0.07–6.49)	0.701
Peritonitis	2	0.54	1.72 (0.05–57.87)	0.751
Meningitis	4	–0.54	0.58 (0.03–9.75)	0.693
Pericarditis	1	dropped	dropped	
**Peripheral Blood**				
QUADAS score				
≥11	8	0.67	1.95 (0.91–4.18)	0.082
<11	11			
Settings				
High TB burden	9	–0.23	0.80 (0.38–1.65)	0.519
Low TB burden	12			
Method				
ELISPOT	13	–0.32	0.73 (0.34–1.58)	0.402
ELISA	8			
Disease				
Pleurisy	12	–1.36	0.26 (0.05–1.32)	0.098
Peritonitis	2	dropped	dropped	
Meningitis	4	–1.99	0.14 (0.02–0.79)	0.029
Mixed*	3	–1.61	0.20 (0.04–1.12)	0.066

^*^Studies contained different extrapulmonary tuberculosis types, including pleurisy, peritonitis, meningitis, and pericarditis.
