# Supplementary material for: Body Fluid Interferon-γ Release Assay for Diagnosis of Extrapulmonary Tuberculosis in Adults: A Systematic Review and Meta-Analysis
Source: Sci Rep. 2015 Oct 27;5:15284. doi: 10.1038/srep15284 (PMC4621514; doi:10.1038/srep15284)
Supplement: Supplementary Information [file srep15284-s1.pdf]

Body Fluid Interferon-γ Release Assay for Diagnosis of Extrapulmonary Tuberculosis in Adults: A Systematic Review and Meta-Analysis

Xiao-Xia Zhou[1, 2 #], Ya-Lan Liu[1#], Kan Zhai[3], Huan-Zhong Shi[1, 3], Zhao-Hui Tong[1,3*]

[1]Department of Respiratory and Critical Care Medicine, Beijing Chao-Yang Hospital, Capital Medical University, Beijing, China

[2]Geriatric Department, Fu Xing hospital, Capital Medical University, Beijing, China

[3]Medical Research Center, Beijing Chao-Yang Hospital, Capital Medical University, Beijing, China

[*]Correspondence: Zhao-Hui Tong, Department of Respiratory and Critical Care Medicine, Beijing Chao-Yang Hospital, Capital Medical University, Beijing 100020, China.

E-mail: tongzhaohuicy@sina.com

[#] These authors contributed equally to the present work.

Supplementary Table S1. Study summary of IGRAs in body fluid

Study	Country	HIV Subjects (%)	Subjects (n)	Disease	IGRA type	IGRA cutoff#	Test results				Quality scores	
							TP	FP	FN	TN	QUADAS	STARD
Zhang *et al.* (2014)	China	0	167	Mixed	ELISPOT	≥6	68	12	6	81	10	19
Liao *et al.* (2014)	China	0	332	Pleurisy	ELISPOT	≥86/100*	268	0	12	51	10	17
Liu *et al.* (2013)	China	0	96	Pleurisy	ELISPOT	≥54	52	3	2	39	12	19
Keng *et al.* (2013)	Taiwan	1.1	52	Pleurisy	ELISPOT	≥6	12	17	2	21	9	16
Kang *et al.* (2012)	South Korea	0	36	Pleurisy	ELISPOT	≥75	19	2	1	14	11	17
					ELISA	≥2.745	12	1	6	14		
Eldin *et al.*(2012)	Egypt	Unknown	38	Pleurisy	ELISA	≥0.35	12	3	8	15	11	16
Cirak *et al.*(2012)	Turkey	Unknown	100	Pleurisy	ELISA	≥0.35	10	23	0	42	9	17
Losi *et al.* (2011)	Italy	0	48	Pleurisy	ELISA	≥3.01	15	14	3	16	10	13
Ates *et al.* (2011)	Turkey	Unknown	67	Pleurisy	ELISA	≥0.30	21	3	4	23	10	17
Cho *et al.* (2011)	South Korea	1.8	50	Peritonitis	ELISPOT	≥14	11	2	1	12	13	19
Patel *et al.* (2010)	South Africa	87.1	86	Meningitis	ELISPOT	≥46	31	3	5	40	9	18
Kim *et al.* (2010)	South Korea	2.3	84	Meningitis	ELISPOT	≥6	13	3	9	25	10	18
Liao *et al.* (2009)	Taiwan	2.9	138	Mixed	ELISPOT	≥10	13	11	4	14	11	19
Lee *et al.* (2009)	Taiwan	0	40	Pleurisy	ELISPOT	≥10	18	3	1	18	11	16
Dheda *et al.* (2009)	South Africa	46.4	56	Pleurisy	ELISPOT	≥24	34	6	6	9	9	18
					ELISA	≥0.35	22	3	19	12		
Chegou *et al.* (2008)	South Africa	64.3	39	Pleurisy	ELISA	≥0.35	13	2	10	13	11	15
Baba *et al.* (2008)	South Africa	73.5	13	Pleurisy	ELISA	≥0.35	3	0	0	3	10	17
Thomas *et al.* (2008)	India, UK, German	0	19	Meningitis	ELISPOT	≥6	9	0	1	7	10	19
Kim *et al.* (2008)	South Korea	5.4	33	Meningitis	ELISPOT	≥20	3	3	1	9	11	19
Losi *et al.* (2007)	Italy, German, Netherland	Unknown	41	Pleurisy	ELISPOT	≥6	19	5	1	16	9	14

Ariga *et al.* (2007)	Japan	0	75	Mixed	ELISA	≥2.352	27	1	1	46	10	19
Wilkinson *et al.* (2005)	UK	0	16	Pleurisy	ELISPOT	≥10	8	1	0	7	10	11
Total			1626									

Unit for cutoff value was SFCs per 2×10^5 cells for ELISPOT and for ELISA

*cut-off value was determined by receiver operating characteristic (ROC) analysis. Eighty-six SFCs for ESAT-6 protein and 100 SFCs for peptide pool.

Supplementary Table S2. Study summary of IGRAs in peripheral blood

Study	Country	Subjects (n)	Disease	IGRA type	IGRA cutoff#	Test results			
						TP	FP	FN	TN
Zhang *et al.* (2014)	China	167	Mixed	ELISPOT	≥6	54	25	20	68
Liao *et al.* (2014)	China	332	Pleurisy	ELISPOT	≥40/30*	220	7	61	44
Liu *et al.* (2013)	China	96	Pleurisy	ELISPOT	≥6	51	16	4	27
Kang *et al.* (2012)	South Korea	36	Pleurisy	ELISPOT	≥21	17	2	4	13
				ELISA	≥0.175	17	5	4	10
Eldin *et al.*(2012)	Egypt	38	Pleurisy	ELISA	≥0.35	14	4	6	14
Losi *et al.* (2011)	Italy	48	Pleurisy	ELISA	≥2.73	14	11	4	19
Ates *et al.* (2011)	Turkey	67	Pleurisy	ELISA	≥0.35	30	10	12	15
Cho *et al.* (2011)	South Korea	50	Peritonitis	ELISPOT	≥21	21	1	7	20
Patel *et al.* (2010)	South Africa	86	Meningitis	ELISPOT	≥48	31	21	6	29
Kim *et al.* (2010)	South Korea	84	Meningitis	ELISPOT	≥6	22	23	9	30
Liao *et al.* (2009)	Taiwan	138	Mixed	ELISPOT	≥10	10	7	2	23
Lee *et al.* (2009)	Taiwan	40	Pleurisy	ELISPOT	≥10	14	2	4	19
Dheda *et al.* (2009)	South Africa	56	Pleurisy	ELISPOT	≥24	30	7	6	9
				ELISA	≥0.35	27	5	4	11
Chegou *et al.* (2008)	South Africa	39	Pleurisy	ELISA	≥0.35	16	5	6	12
Baba *et al.* (2008)	South Africa	13	Pleurisy	ELISA	≥0.35	7	0	1	5
Thomas *et al.* (2008)	India, UK, German	19	Meningitis	ELISPOT	≥6	9	2	2	6
Kim *et al.* (2008)	South Korea	33	Meningitis	ELISPOT	≥6	9	9	0	15
Losi *et al.* (2007)	Italy, German, Netherland	41	Pleurisy	ELISPOT	≥6	18	7	2	14
Ariga *et al.* (2007)	Japan	75	Mixed	ELISA	≥0.281	21	14	6	33
Wilkinson *et al.* (2005)	UK	16	Pleurisy	ELISPOT	≥6	8	0	Nil	Nil

Unit for cutoff value was SFCs per 2×10^5cells for ELISPOT and IU/ml for ELISA

*cut-off value was determined by receiver operating characteristic (ROC) analysis: 40 SFCs for ESAT-6 protein and 30 SFCs for peptide pool.

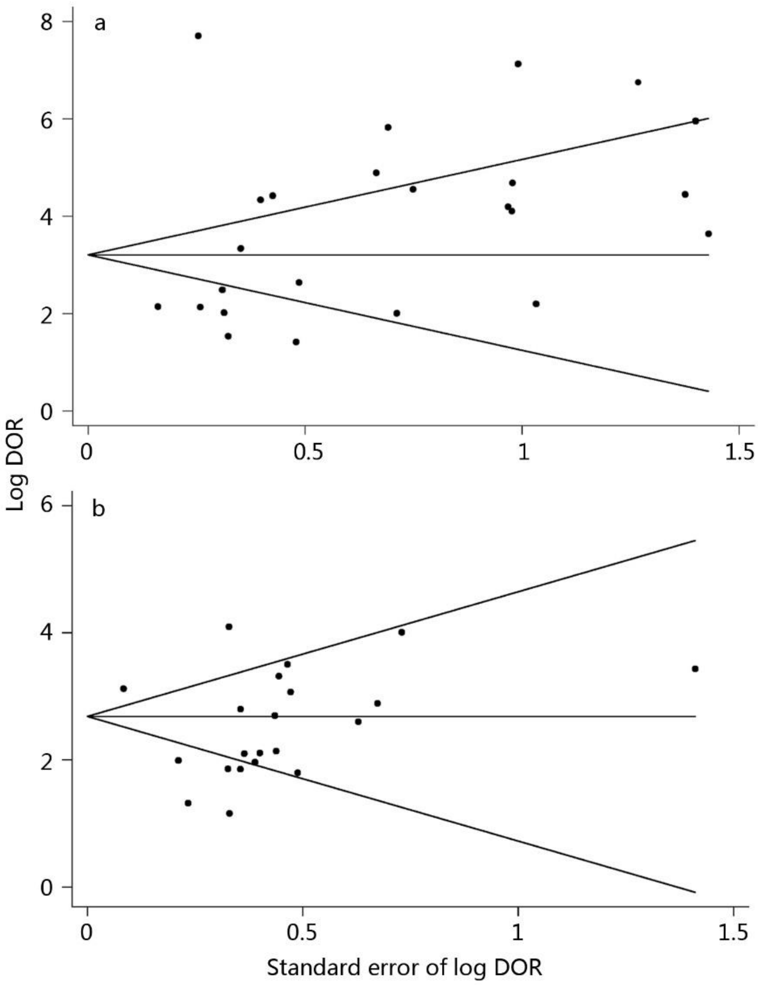

Supplementary Figure S1. Funnel graph for the assessment of potential publication bias in studies of IGRAs in body fluid (a) and peripheral blood (b). Solid circles indicate each study in the meta-analysis. The central lines represent the summary diagnostic odds ratio (DOR).
